# Landscape Alteration by Pre-Pottery Neolithic Communities in the Southern Levant – The Kaizer Hilltop Quarry, Israel

**DOI:** 10.1371/journal.pone.0150395

**Published:** 2016-03-09

**Authors:** Leore Grosman, Naama Goren-Inbar

**Affiliations:** Institute of Archaeology, Mount Scopus, The Hebrew University of Jerusalem, Jerusalem, 91905, Israel; New York State Museum, UNITED STATES

## Abstract

This study focuses on Kaizer Hill, a quarry site located in the vicinity of the city of Modiin where remains of a single prehistoric cultural entity assigned to the Pre-Pottery Neolithic A were discovered. A systematic survey revealed that large-scale quarrying activities have left damage markings on the bedrock of the Hilltop and its slopes. We aim to present here our findings from the Hilltop, which are concerned with the human impact on rock surfaces and the lithic artifacts retrieved during the survey. It is evident that the Pre-Pottery Neolithic A inhabitants of the area changed their landscape forever, “stripping” the *caliche* surface and penetrating it in search of flint bedded in the bedrock.

## Introduction

The Neolithic Pre-Pottery Neolithic A (PPNA) culture, dated from 11,600 to 10,200 calBP [1], is one of the incipient cultural stages in the shift to agricultural subsistence, which in the following periods would replace the mode of hunting and gathering. Recent studies in the southern Levant have shown that this economic shift was accompanied by numerous changes in the social and technological spheres associated with remarkable evidence of developments in, among others, architecture [2], sedentism [3] and pyrotechnology (e.g. [4]). In addition, Grosman and Goren-Inbar [5] have recently presented a new interpretation of bedrock cupmarks located in the vicinity of the PPNA site of Hatula. This area contains evidence of quarrying activity aimed at extracting flint nodules and exploiting the thick layer of *caliche* (locally known by the Arabic term *nari)*. This suggestion differs from the commonly held view, which considers all features defined as cupmarks to be devices that were primarily involved in a variety of grinding, food preparation, social or even symbolic activities (e.g., “bedrock features” in [6]).

The Hatula cupmark site was the first Levantine PPNA quarry site to be identified. Previously, Lower Paleolithic quarry sites [7–9] and a single Middle Paleolithic occurrence [10–12] have been reported. Late Neolithic quarries are known from the Pre-Pottery Neolithic B [13, 14] including the recently reported remarkable larnite quarry [15]. The study of the Hatula PPNA landscape revealed, in addition to cupmarks, an array of features related to the extraction of slabs of limestone (*caliche*). It was further suggested that drilling was the primary technique for extracting flint nodules from the *caliche* at Hatula [5].

The main question that rises from the above discoveries is whether the Neolithization process initiated a novel approach to the landscape. Was Hatula a unique phenomenon or can a different scenario be reconstructed? Were Neolithic communities actively involved in transforming their landscape?

**Fig 1:**
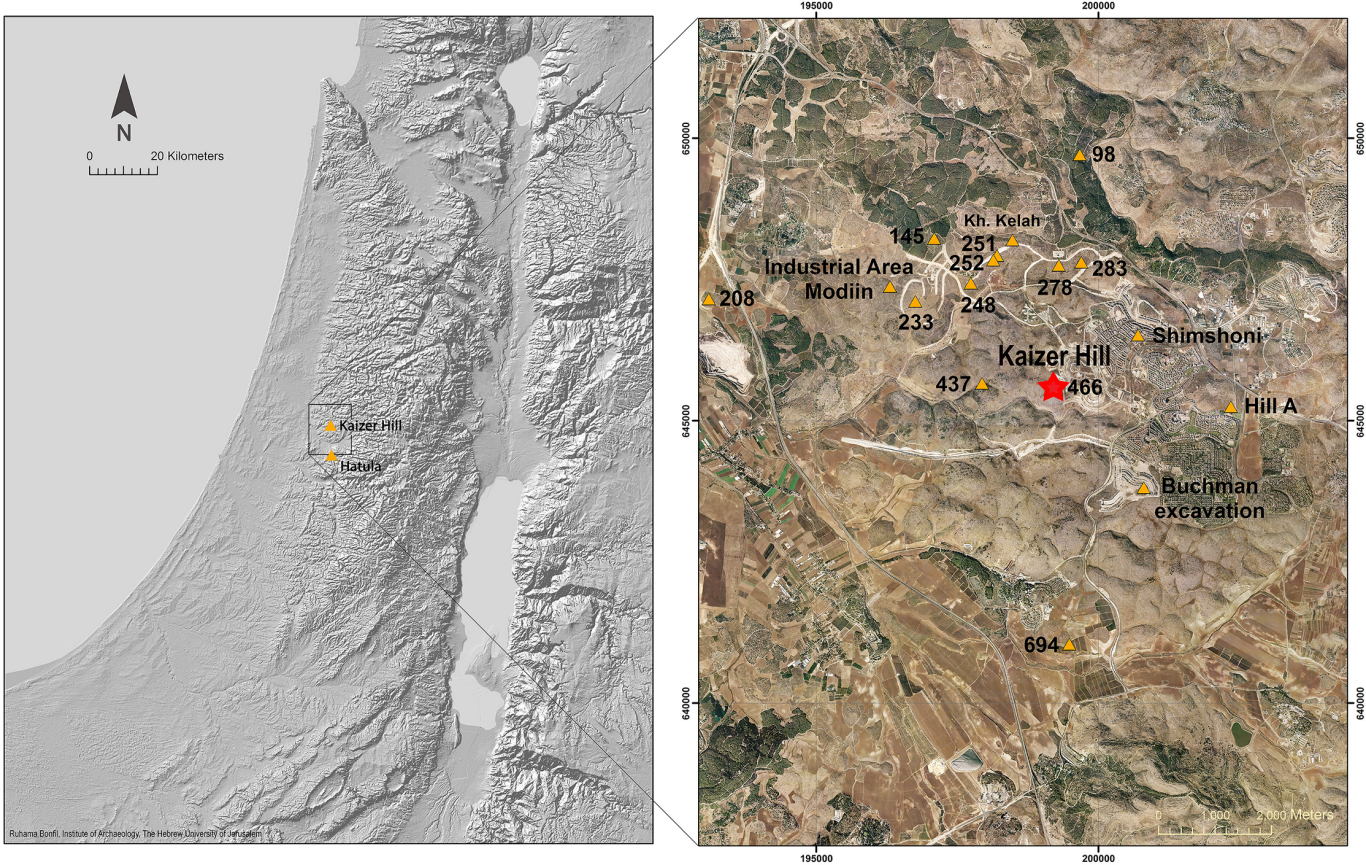
Map of PPN sites (Δ) in the vicinity of Kaizer Hill.

Thus, the observations from the Hatula project and the hypothesis that derived from them required additional testing and verification. Consequently, a search was conducted in the vicinity (ca. 10 km radius, Fig 1) with the aim of establishing whether similar geological settings reveal PPNA quarrying activities resembling those identified at Hatula. The main guideline in our search was the *caliche* surface that was the preferred rock for the quarrying activity at Hatula. Other studies carried out on hills in the vicinity of Hatula resulted in the identification of rock damage markings at, e.g., Tzur Natan [16], Modiin Buchman, Nahal Nevallat ([17] and references therein) and many others (Fig 1).

**Fig 2:**
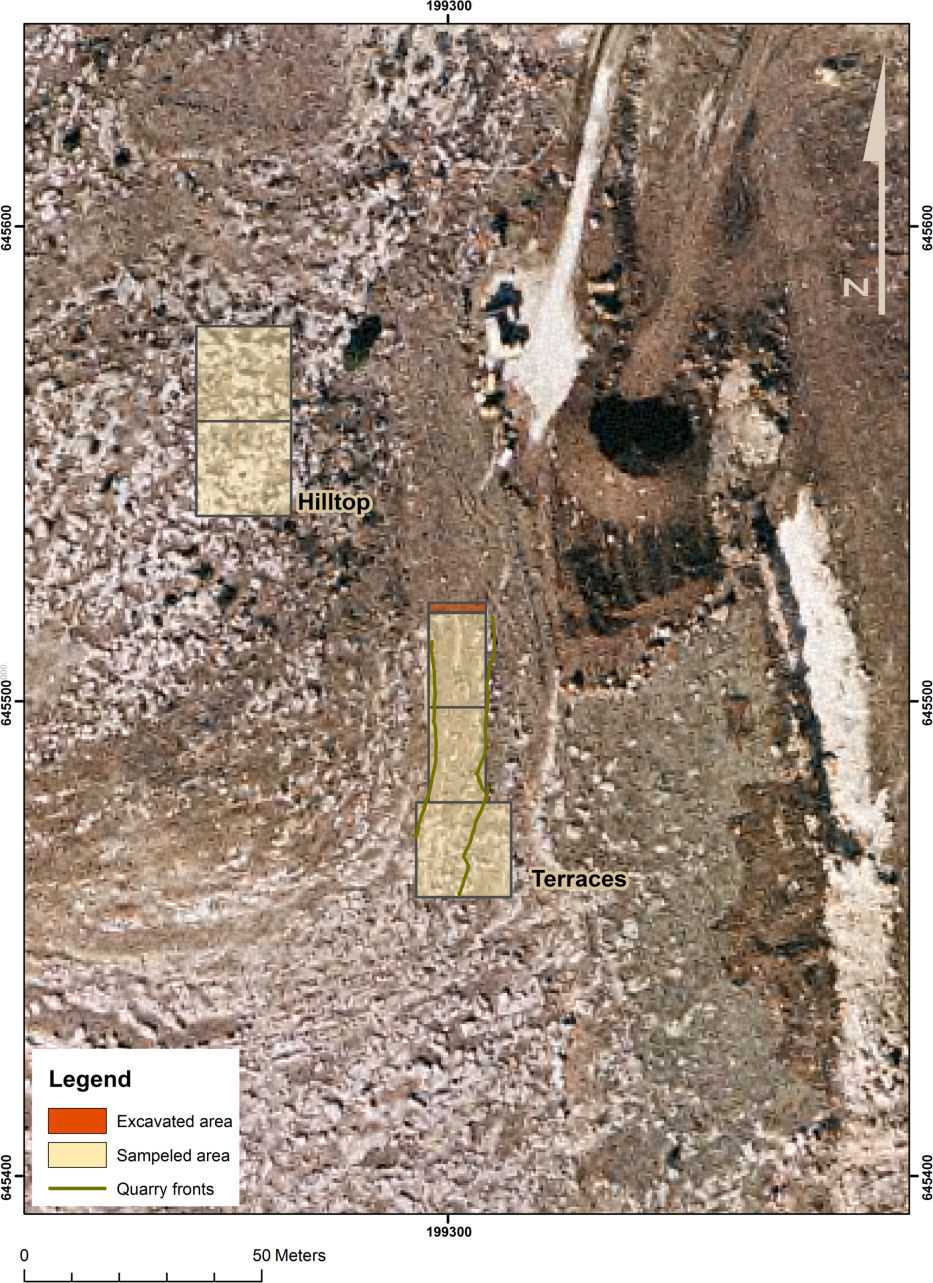
Aerial view of Kaizer Hill showing studied area.

We decided to focus our investigations on Kaizer Hill, located in the vicinity of the city of Modiin (Fig 1), as a previous survey conducted by the Israel Antiquities Authority had identified a single PPNA site there. This identification was based on small lithic assemblage, part of which was left in the field, retrieved from the survey and from excavation of a limited area confined to the Hilltop (not overlapping the area surveyed in the present study) [18]. During this survey several cupmarks were identified on the bedrock of Kaizer Hill and its vicinity, although their nature or function was not specified. Our preliminary survey revealed a high concentration of waste piles, cupmarks and quarry fronts that required in-depth investigation. Following these results we initiated our study (2007–2009) at Kaizer Hill, some 7 km as the crow flies from Hatula. Topographically, Kaizer Hill is divided between the Hilltop, composed of *caliche*, and the terraced slopes (Fig 2), composed of hard limestone dotted with flint. These slopes were formed by a series of terraces, which we consider to result from extensive quarrying activities that are beyond the scope of the present study.

The large-scale bedrock damage markings identified on the hilltop and its slopes necessitated an extensive study [19, 20]. The present contribution, however, is concerned only with the Hilltop due to its complex nature and unique characteristics, which differ markedly from those of the terraces.

We aim to present here our findings, which are concerned with the human impact on the rock surfaces that is reflected by an array of rock damage markings that have survived erosional processes. The findings include a description of the various damage patterns and the lithic assemblage retrieved from the Hilltop quarry area. This account will facilitate comparison between Kaizer Hill and the Hatula quarry site, as well as other PPNA sites, and will hopefully result in the identification of behavioral patterns, tactics and strategies adopted by the Neolithic groups in the process of “domesticating” the landscape.

### Location

Kaizer Hill is located in the southwestern outskirts of Modiin, a city that is expanding rapidly with massive construction and hence also destruction of the hills that immediately surround it. It is part of the series of low hills located on the border between the Shephelah and the Judean Hills at about 250 m above msl (Fig 1). The hills and wadis of this region are characterized by eroded sedimentary rocks associated with karstic phenomena. Quarrying activities on the bedrock in the area are recorded since historical times and remains from as early as the Neolithic period are documented (e.g., [16, 18, 21–25]).

### Geology

**Fig 3:**
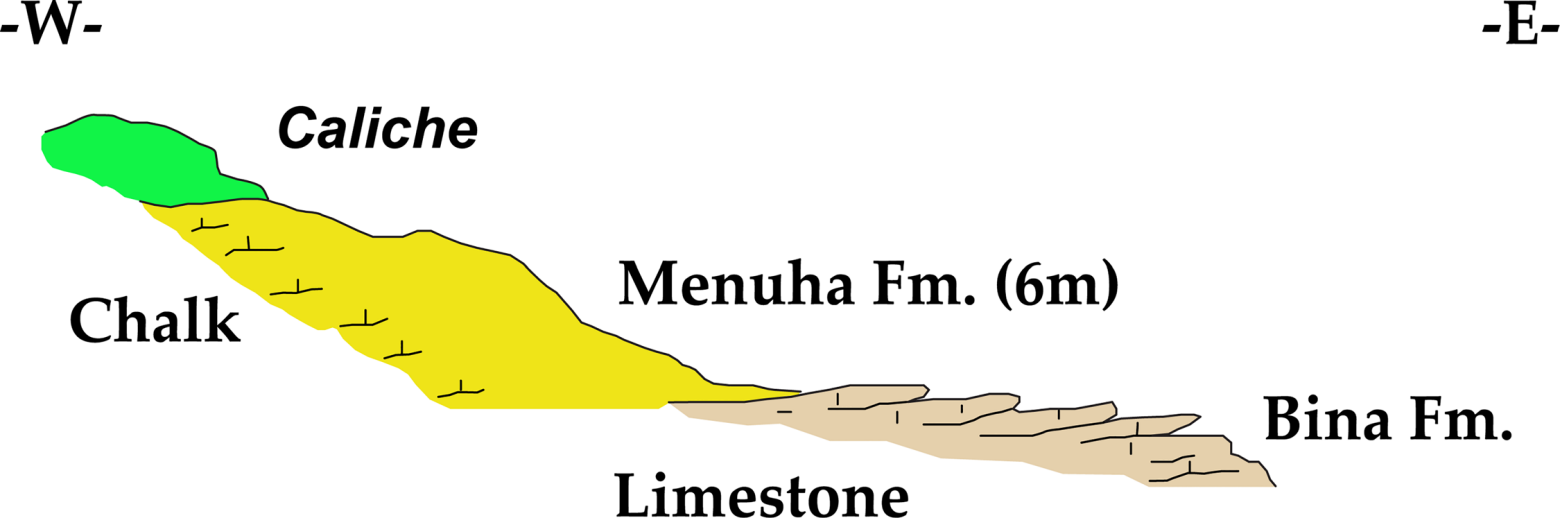
East-west geological section of Kaizer Hill (drawn by A. Starinsky).

Kaizer Hill is composed of two geological formations (Fig 3): a) the topographically lower Bina Formation (Turonian), consisting mainly of well-bedded hard limestone, and b) the upper Menuha Formation (Santonian), made of massive chalk [26]. This chalk is capped by a well-developed typical *caliche* (maximal thickness 1.5 m). Both formations include bedded flint. The *caliche* contains small to large sharp-edged brown flint fragments typical of the Meshash Formation bedded in it. These flint fragments are residual feature of the once overlying flint-rich Meshash Formation (Santonian Campanian), which has been completely eroded from Kaizer Hill; the flint represents a paleo-surface relict that predates the formation of the *caliche* [26].

Markings on bedrock were identified on both bedrock surfaces (*caliche* and Bina Formation).

Repeated visits to Kaizer Hilltop, followed by three very short field seasons, resulted in the identification of densely scattered damage markings on the *caliche* rock surfaces.

## Methodology

### The sampled rock surfaces

**Fig 4:**
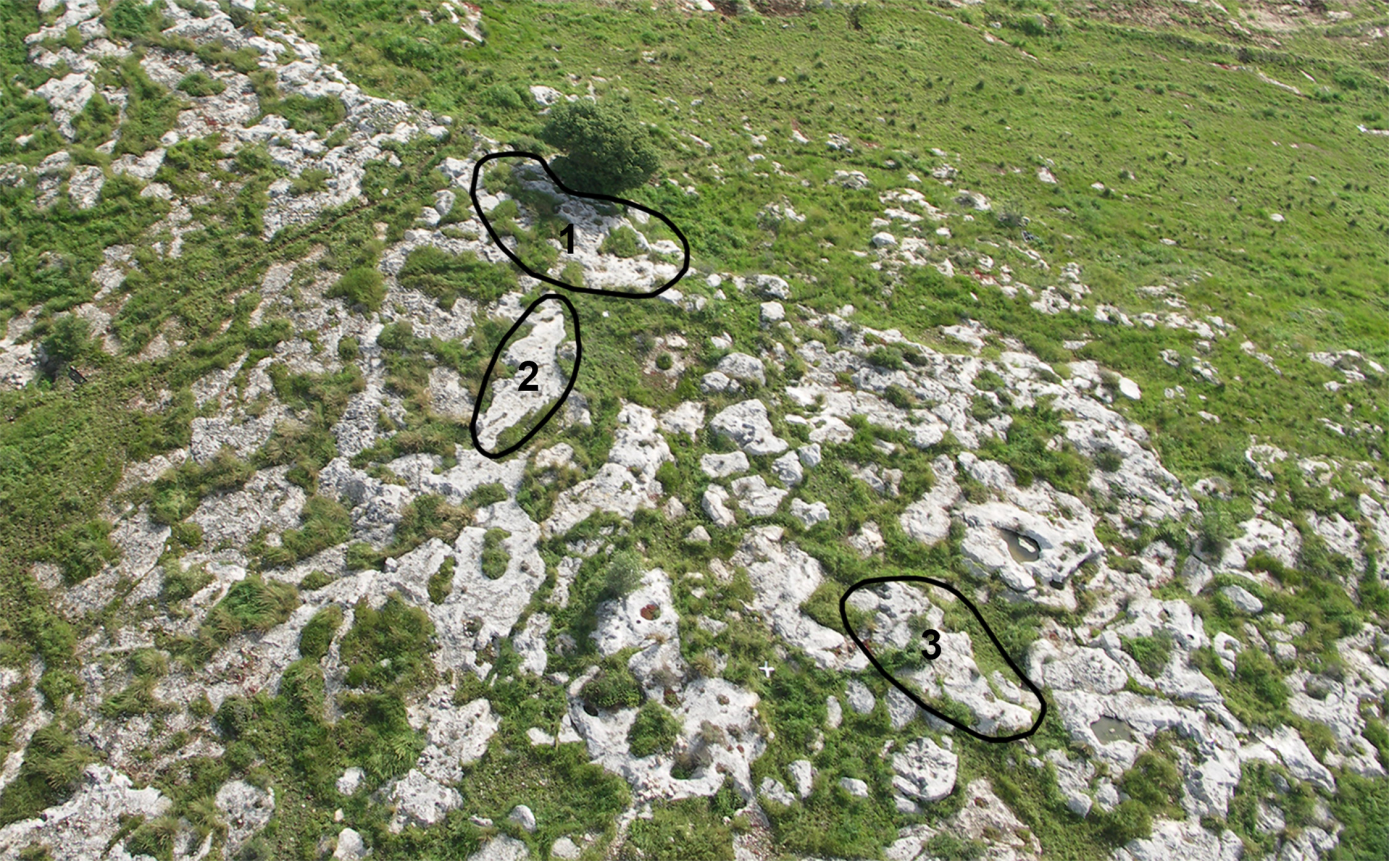
Aerial view of the Hilltop and the three sampled rock surfaces.

**Fig 5:**
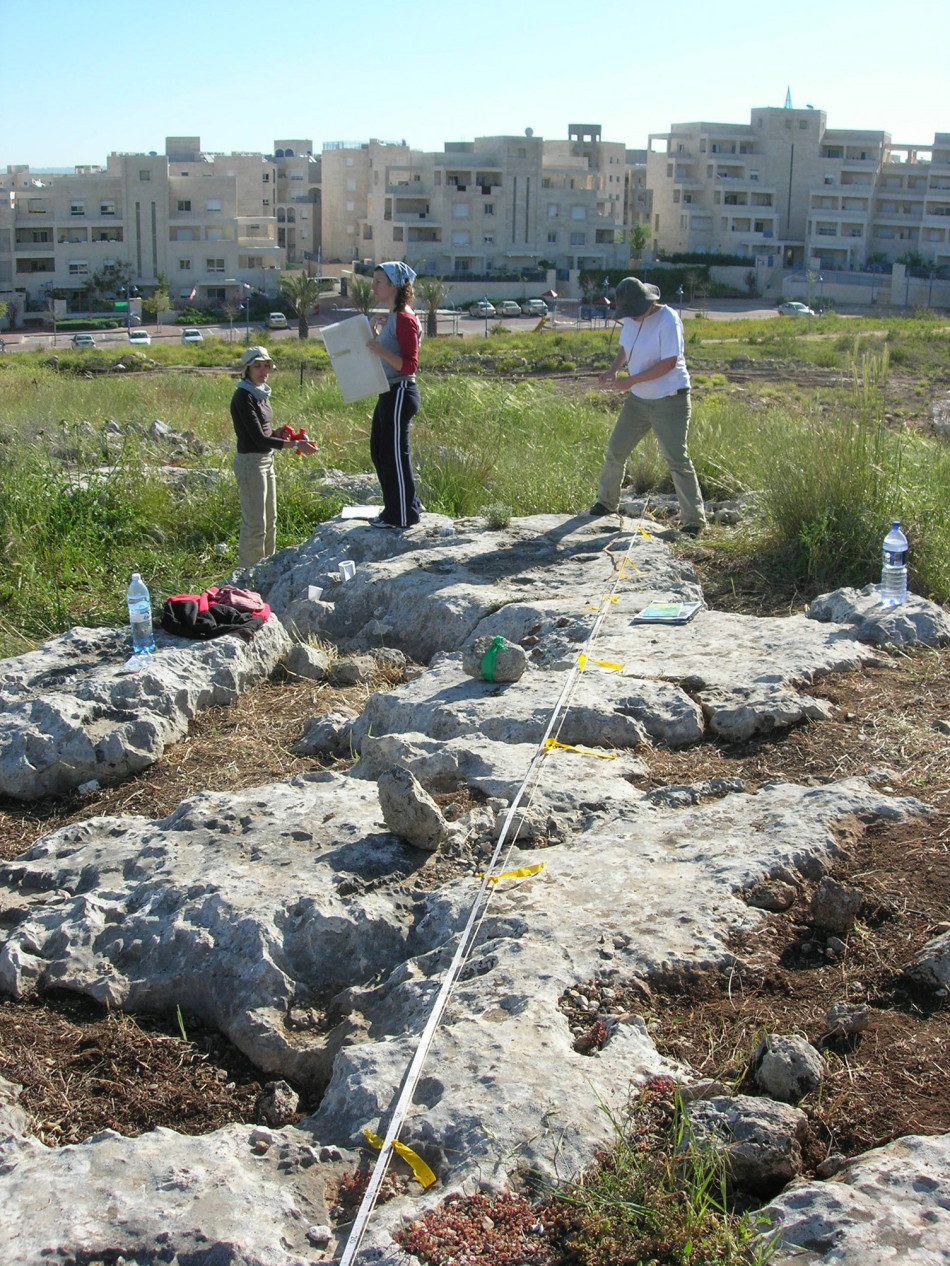
Rock #3 and the team at work.

The first step of the survey was to identify the range of rock damage types reflecting quarrying activity on the hilltop. This was accomplished by selecting for detailed study three *caliche* rock surfaces bearing a variety of rock damage types, including cupmarks and quarrying fronts. The surfaces, located topographically one above the other on the slope of the Hilltop, were designated Rocks #1, #2 and #3 (Figs 4 and 5). The analyses incorporated photography and drafting of detailed plans on which the precise locations of the various rock damage features were marked (Israel Antiquities Authority Permits G-36/2007, G-29/2008, G-41/2009; our study does not require an ethics statement; the individual in figures has given written informed consent to publish these case details).

In order to achieve objective, accurate and detailed documentation, a type list that included a detailed attribute list of the various types of bedrock damage was generated. It aimed to describe the different markings observed and involved a division into four main categories of rock damage. These categories are: A) cupmarks; B) “trenching” features formed by a series of drilling marks arranged in a row and forming a line ([5]with description and illustrations therein); C) various rock damage markings that resulted from the application of tools to the *caliche* surface; D) quarry fronts. The recorded attributes generated a database that contains the complete information for each of the three studied rock surfaces.

### The Flint Assemblage

During the survey of rock surfaces systematic collection of flint artifacts was carried out, sampling a surface of 853.5 m^2^ (all recovered materials are curated at the Institute of Archaeology, at the Hebrew University of Jerusalem). Sampling was divided into two designated areas:

- One meter around the selected rock surfaces, primarily adjacent to the quarry fronts: Rock #1–12.5 m^2^, Rock #2–24 m^2^ and Rock #3–17 m^2^.
- Total surface collection of all lithic artifacts from two squares each measuring 20 x 20m (800 m^2^) in close proximity to the summit of the Hilltop (Fig 2).

Detailed techno-typological lithic analysis of the flint artifacts was conducted by means of an attribute analysis methodology that is commonly applied to PPNA lithic assemblages [27–30].

## Exploring the Caliche

**Fig 6:**
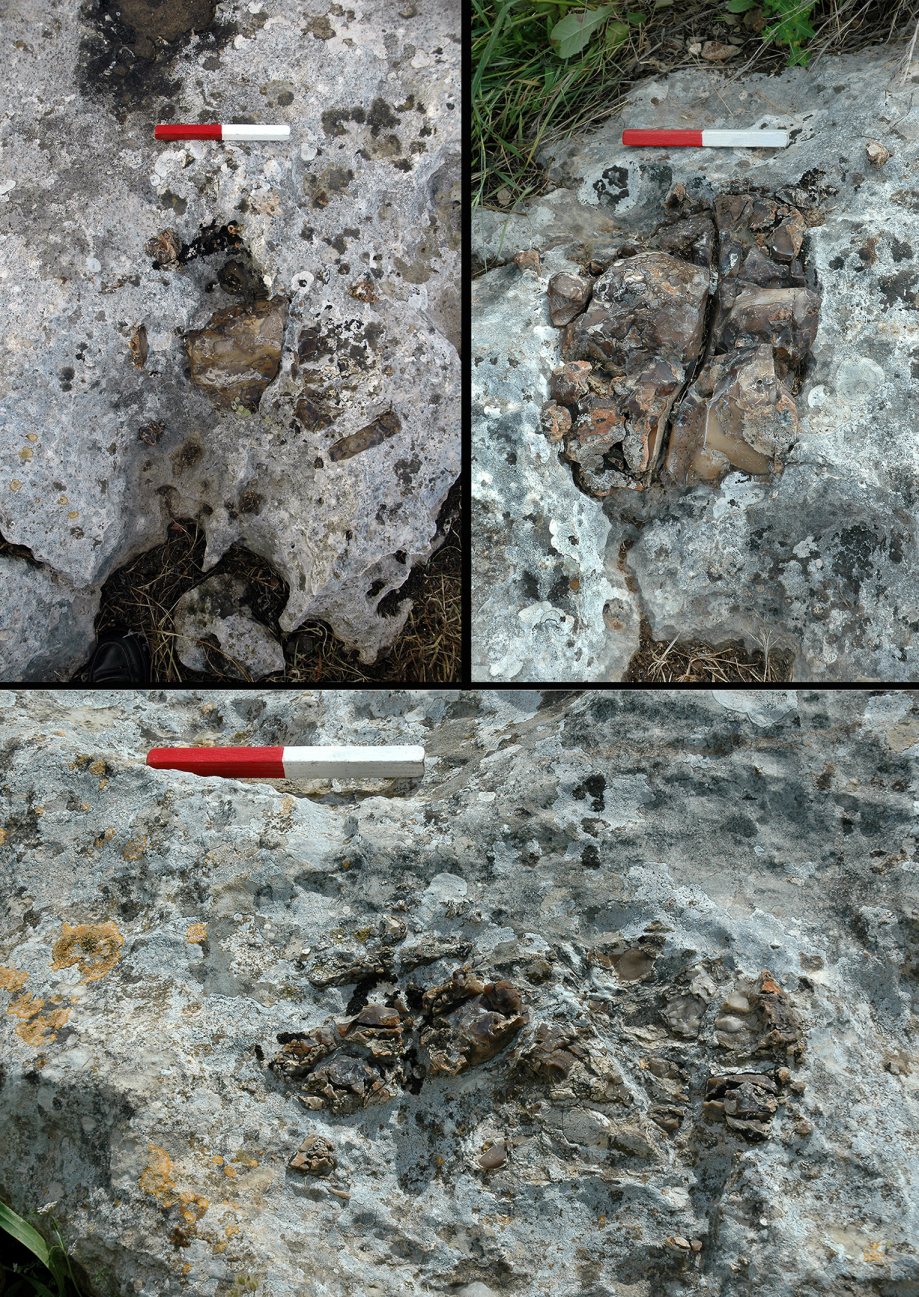
Three views of fractured Meshash Formation flint bedded in the *caliche* crust (length of scale = 20cm).

Detailed observation of the three rock surfaces of Kaizer Hilltop resulted in the identification of flint boulders and nodules bedded in the *caliche* layer (Fig 6A–6C). The density and size of the natural flint fragments on the Hilltop vary substantially and they are only minimally visible. Nonetheless, fragments can be seen integrated within the 0.50 cm thick *caliche* crust, mainly on some eroded slopes of the *caliche*. This situation differs from descriptions of other hill sites surveyed in the vicinity of Modiin, where the Meshash Formation forms uniform continuous beds that are much better preserved than its residual appearance at Kaizer Hilltop (e.g., [21], and see photograph therein).

During the survey it was noted that several exposed flint inclusions were small in size and were not exploited, and hence the *caliche* surfaces lack the damage markings so typical of other parts of Kaizer Hill. Despite this, some flint nodules were left exposed in situ in the *caliche*, with some markings in close proximity (Fig 6A–6C). These nodules are highly fractured/fissured and are consequently unsuitable for knapping.

The total studied surface of the three selected rock surfaces is 30 m^2^ (Table 1).

**Table 1:** Size of rock surfaces and frequencies of lithic artifacts.

	Rock surface (in m^2^)	Cupmarks (N)	Flint artifacts (N)
Rock #1	6.5	54	96
Rock #2	16	46	832
Rock #3	7.5	36	80
Total	30	136	908

The following presentation provides the results of the typological study of the rock damage markings and its documentation for each of the three studied rock surfaces.

### Cupmarks

**Fig 7:**
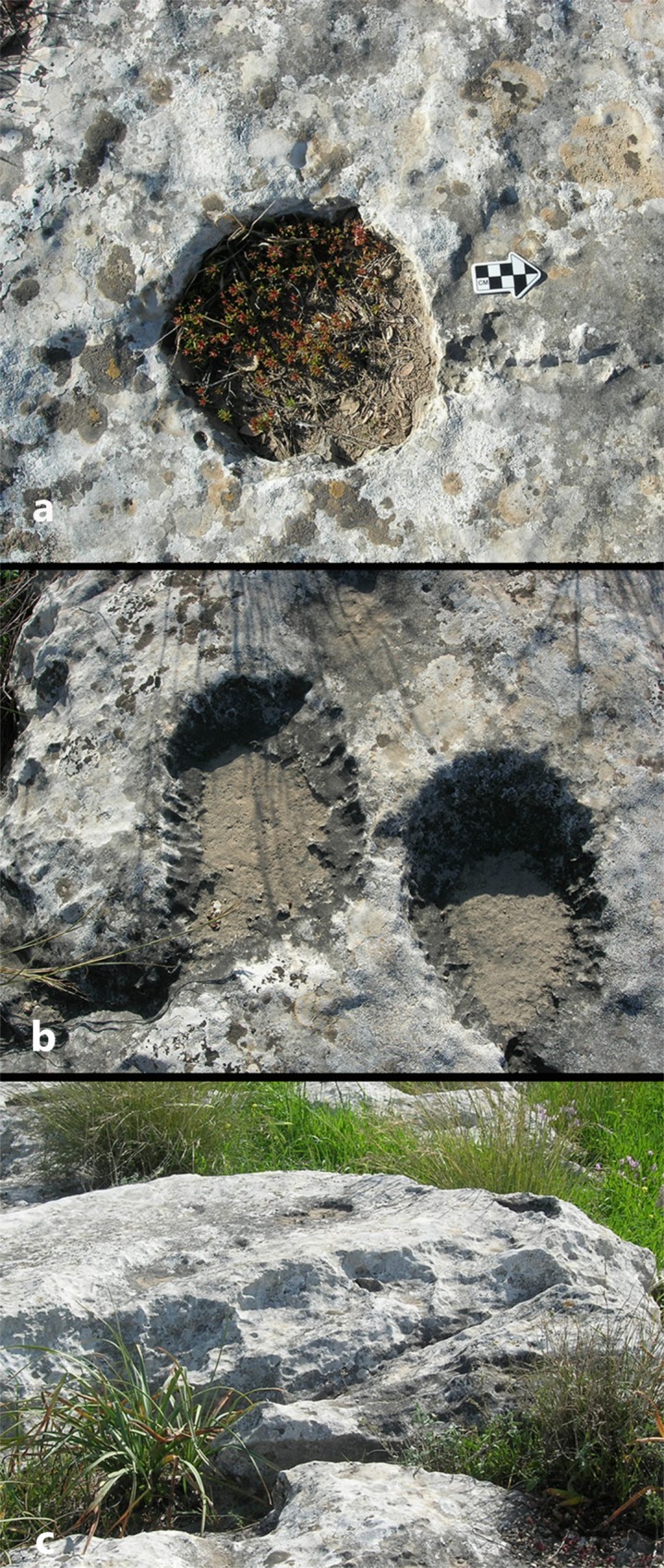
Cupmark contour types: a) rounded; b) “sandal”; c) partial.

**Fig 8:**
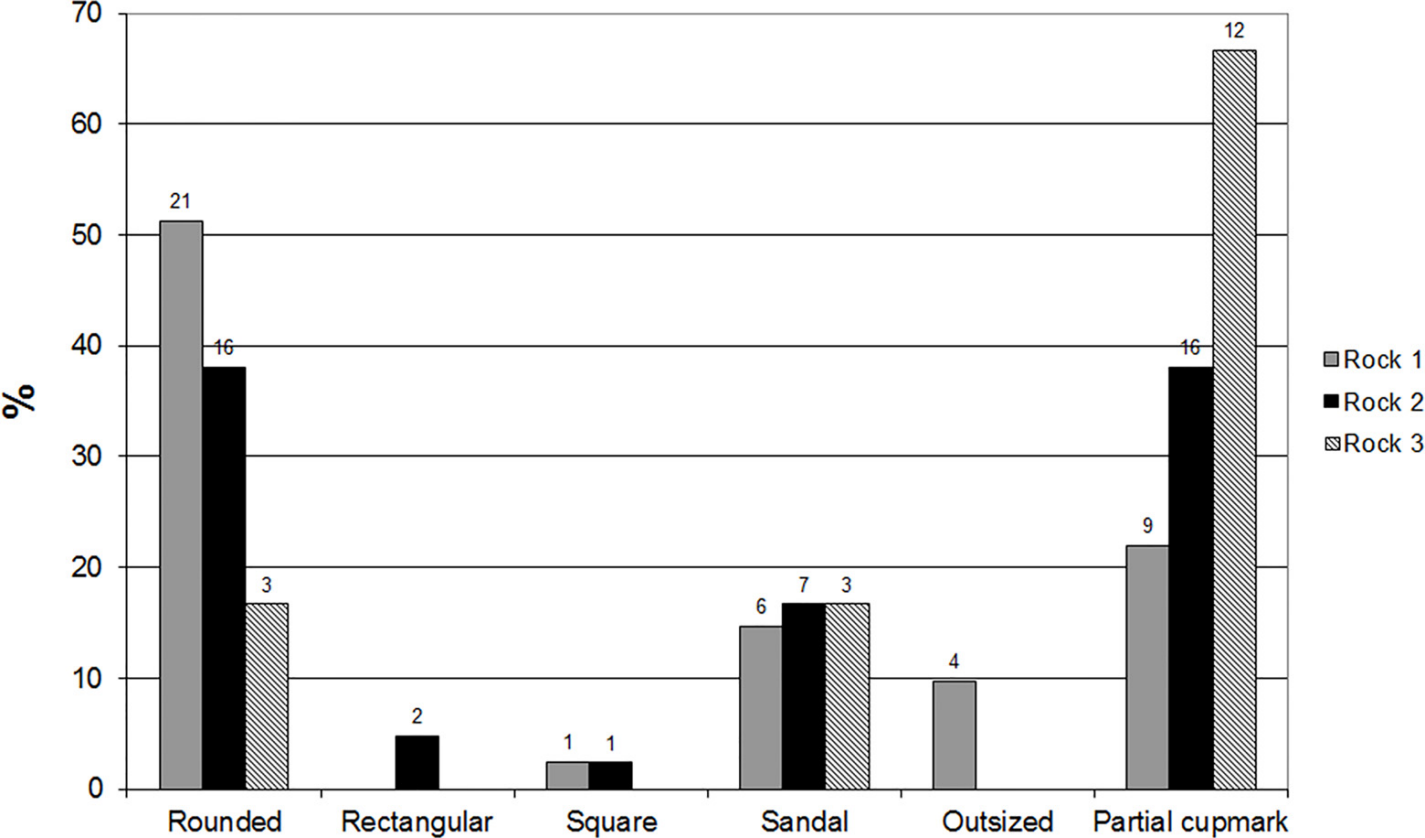
Types and frequencies observed on the three sampled rocks.

**Fig 9:**
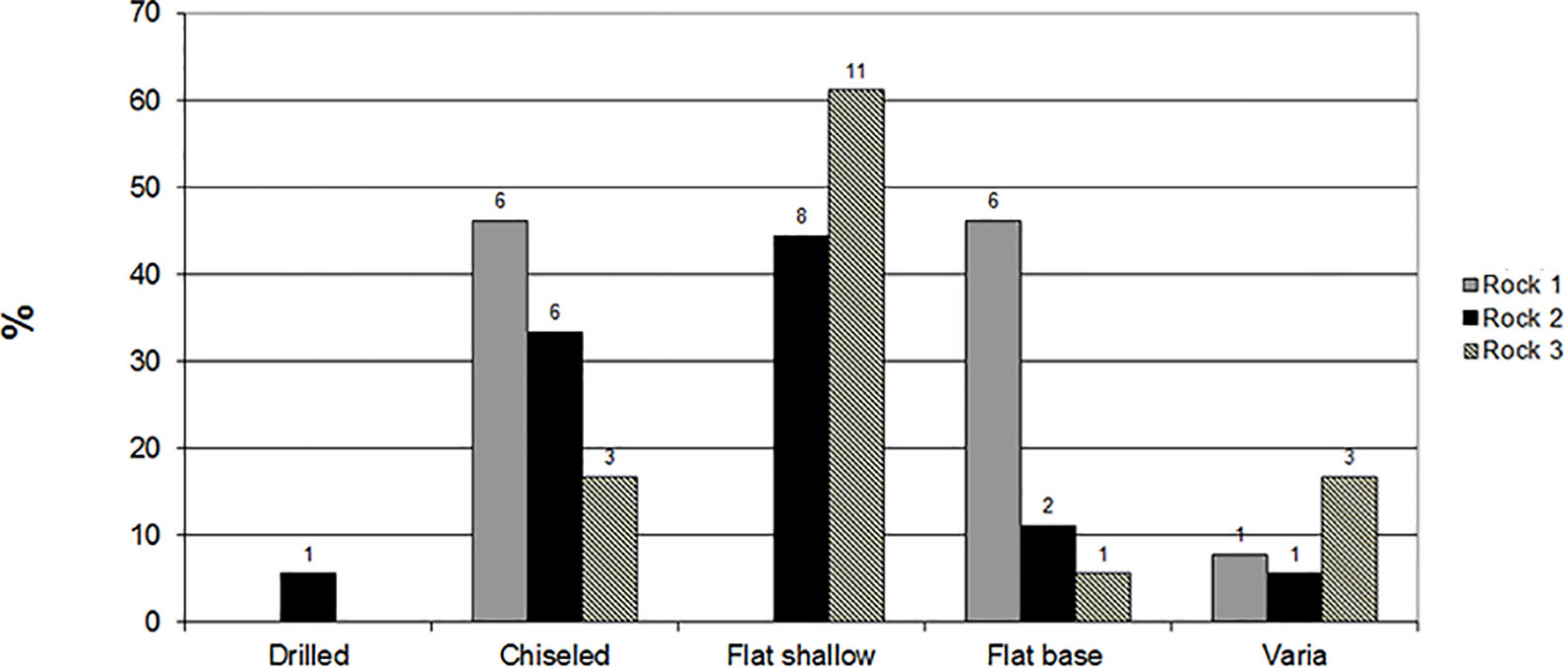
Technological characteristics and frequencies observed on the three sampled rocks.

We documented a variety of cupmarks and identified six contour types observed in the field: rounded (Fig 7A), rectangular, square, “sandal” (Fig 7B), outsized and partial (Fig 7C). The “sandal” type is based on a commonly used term for oblong-oval, shallow, wide, rut-like grooves with flattened bottom and sloping edge (Fig 7B). These are considered to be a typical trait of the Chalcolithic period [17, 21], although it should be noted that no Chalcolithic finds have been retrieved from either Hilltop or terraces of Kaizer Hill. Figs 8 and 9 provide the typological and technological details of the cupmarks observed on the three sampled rock surfaces.

**Fig 10:**
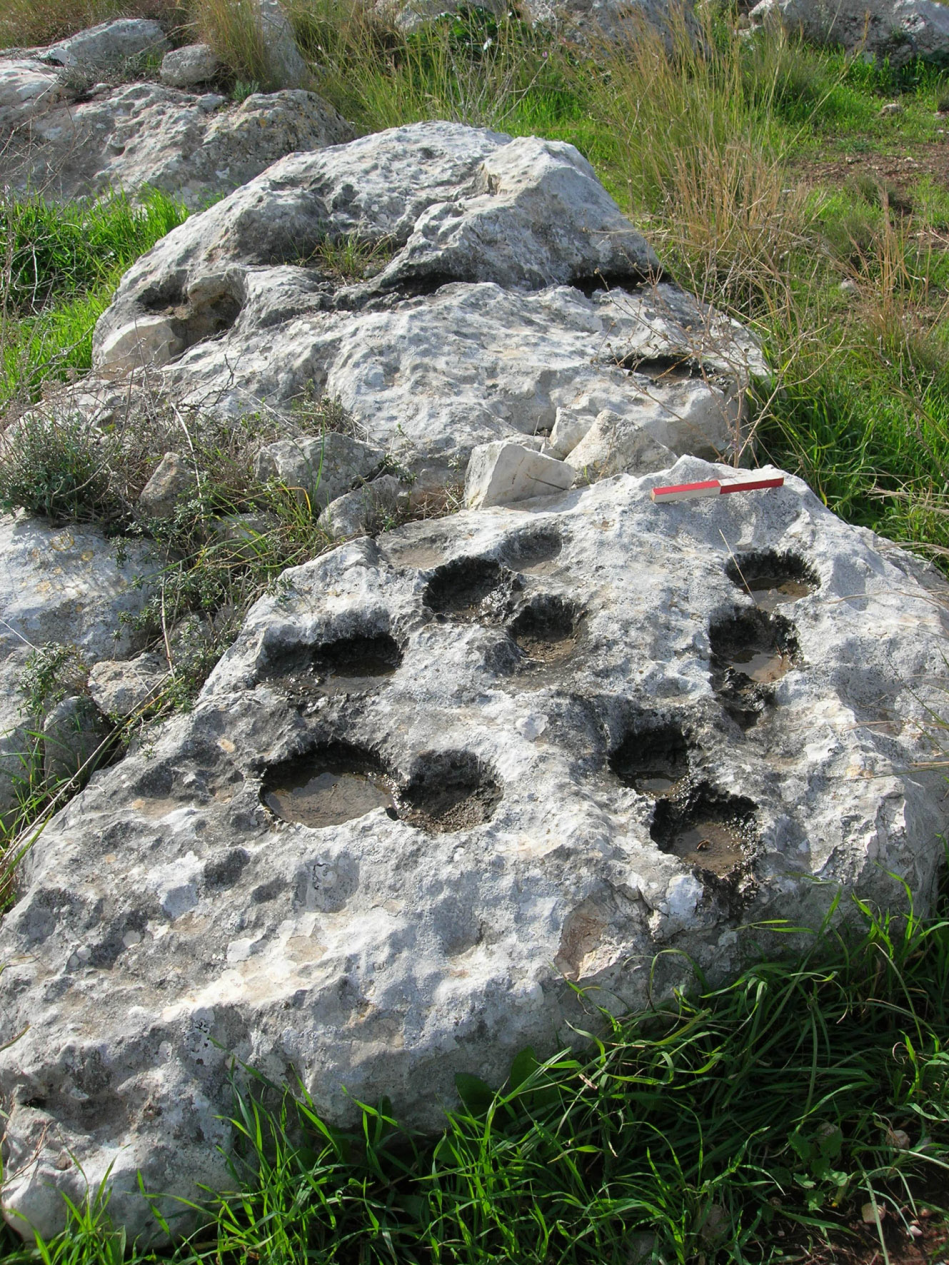
A cluster of cupmarks (length of scale = 20cm).

The most common contour type on the three surveyed rocks surfaces is partial, representing cupmarks that were originally rounded, but continued extraction of the *caliche* has left only a partial contour (Figs 7C and 8). For example, on Rock #3 more than 65% (N = 16) cupmarks are partial and the rest are of the rounded and “sandal” types (each N = 3). Next in frequency are rounded cupmarks, which seem to result from the very common technique of repeated exploring (“opening”) of the rock surface (Fig 10). After this comes the “sandal” type, with similar frequencies on all the sampled rock surfaces. Only a few rectangular and squares cupmarks were recorded. Worth noting are the very large cupmarks (“outsized”) on Rock #1, registered only on this sampled rock surface.

Cupmarks were further divided into four sub-groups on the basis of their technological characteristics: “drilled”–cupmarks that have a small drilled cavity in their bases (ca. 1 cm in diameter); “chiseled”–cupmarks that have long, parallel markings (2–3 cm wide) along their walls (Fig 7B); “flat-based”–relatively deep cupmarks with flat bottoms; and “shallow-based”–cupmarks with walls of minimal height. A high frequency of shallow-based cupmarks was observed on Rocks #2 and #3 and none were observed on Rock #1. In contrast, there are many flat-based cupmarks on Rock #1. All three rock surfaces have chiseled cupmarks in various frequencies, but only Rock #2 has a few drilled cupmarks.

**Fig 11:**
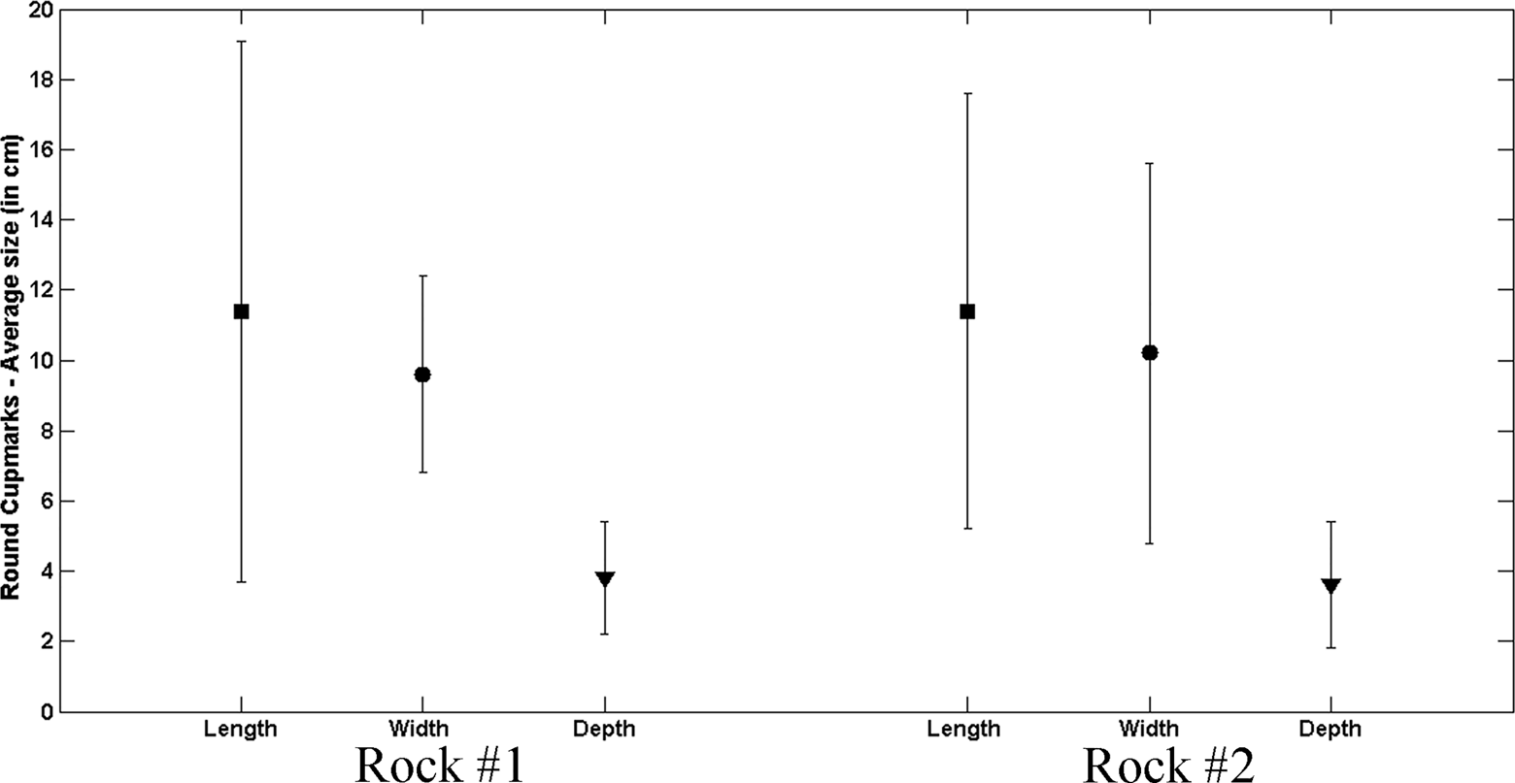
Average size of round cupmarks on Rocks #1 and #2.

The size measurements recorded for each cupmark (length, width and depth) present somewhat similar values but have a high standard deviation: for example, the length average of round cupmarks is ca. 11 cm, while the standard deviation is 7.7 cm (Fig 11). We interpret this very high size variability as resulting from great differences in the sizes of the embedded flint and also to the diverse strategies that were applied in order to extract the flint.

### Trenching (drilling)

**Fig 12:**
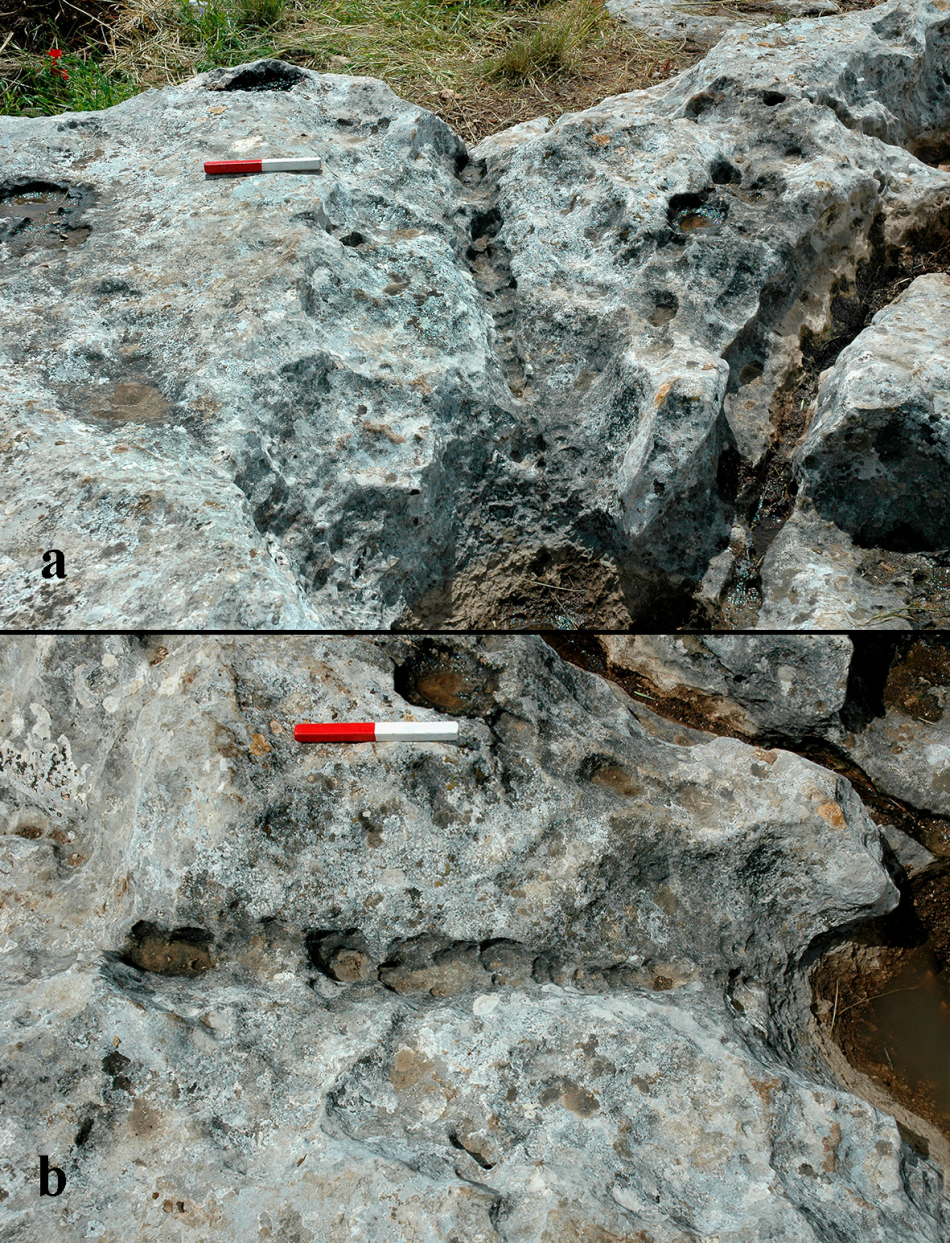
Trenching marks: a) vertical b) horizontal (length of scale = 20cm).

There is clear evidence that drilling technology was known and widely practiced by the PPNA occupants of Kaizer Hill. At Kaizer Hilltop most of the drill markings are located not at the base of the cupmark but perpendicularly to the surface, on the slopes of the quarrying fronts or aligned with the rock surfaces. Drilling marks are visible in various forms: scattered, aligned on the surface to form linear trenching, or vertical, multi-surface lines adjacent to one another and thus forming a long and wide trench (Fig 12). We assume that the main purpose of this trenching was to test the bedrock and locate areas where raw material was abundant and of suitable size for knapping. In addition, drilling was used as a technology for dividing the large rock mass into smaller segments, making the flint caches more easily visible.

**Fig 13:**
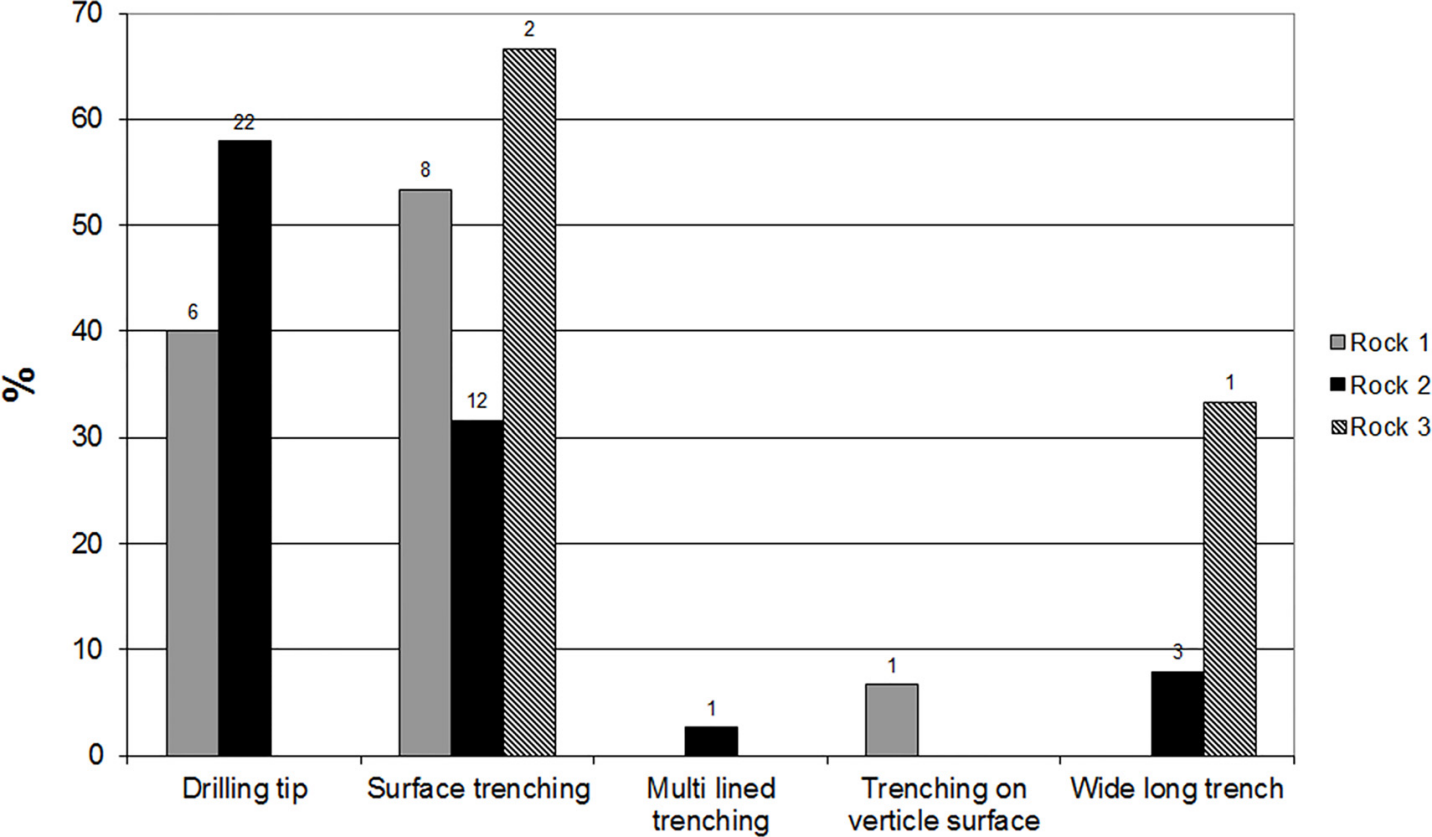
Types and frequencies of trenching marks.

Fig 13 presents the frequencies of trenching rock damage on the three rock surfaces. The most common modes used for trenching are “single drillings” and of “surface trenching”. One rarely finds these methods beside the “long, wide trenches” that was observed in Rock #3.

### Quarrying fronts

Where the *caliche* crust was damaged by quarrying activities, its appearance is one of slightly sloping flat surfaces with discernible boundaries. These boundaries, which are located at the edges of the rock surfaces, are formed by topographic drops to the bottom of the *caliche* crust. This particular and unique topography of drops in height is not a natural feature but resulted from activities that caused extensive rock damage. Our survey showed that whenever these two features (flat surfaces and topographic drops) coexist, the sloping or vertical walls occur where the *caliche* crust is the thickest (ca. 1.5 m) on the Hilltop. In areas where quarrying did not occur, there are moderate slopes with no vertical *caliche* wall.

**Fig 14:**
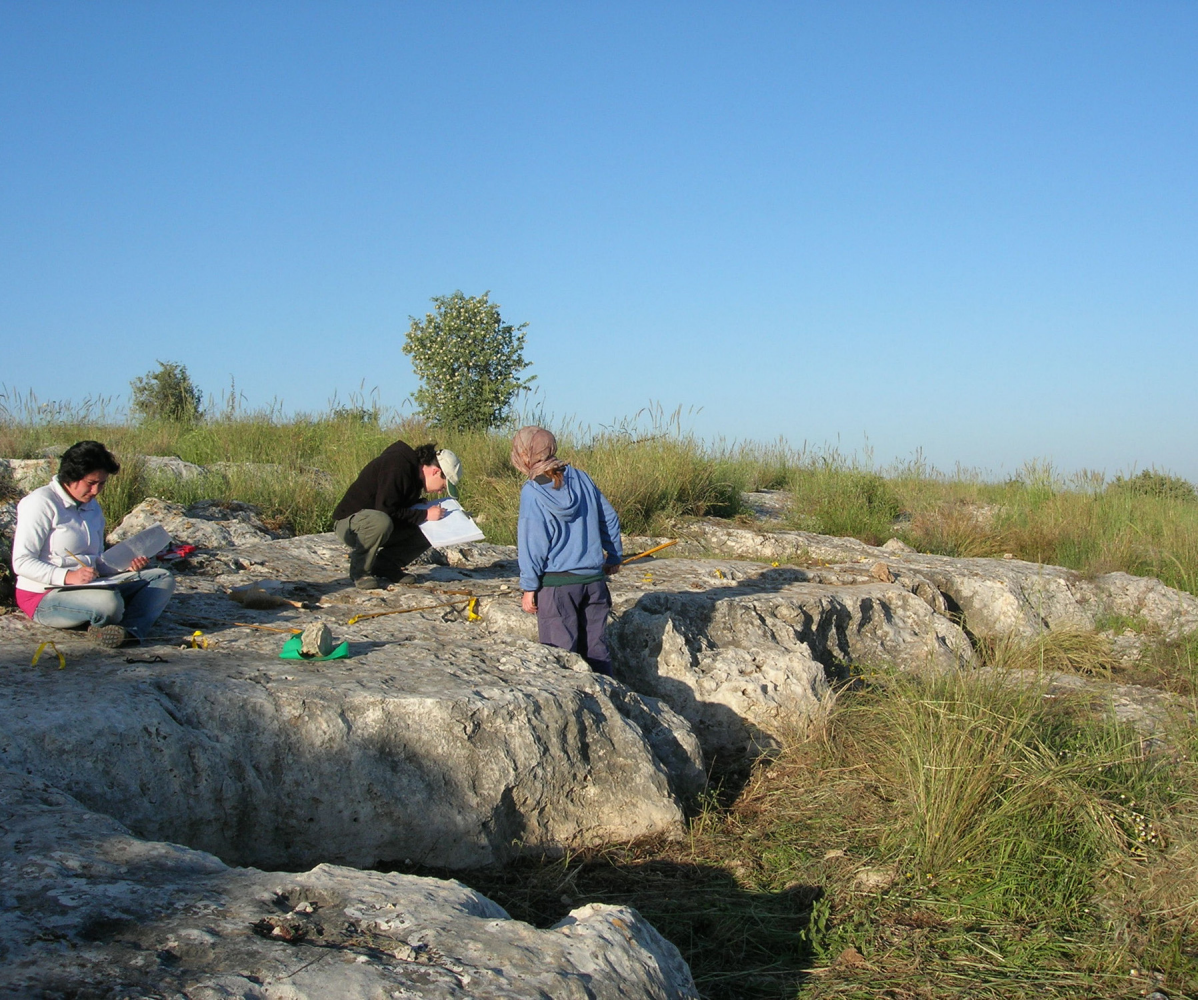
Quarrying front: note the flat top surface and the non-linear edge.

**Fig 15:**
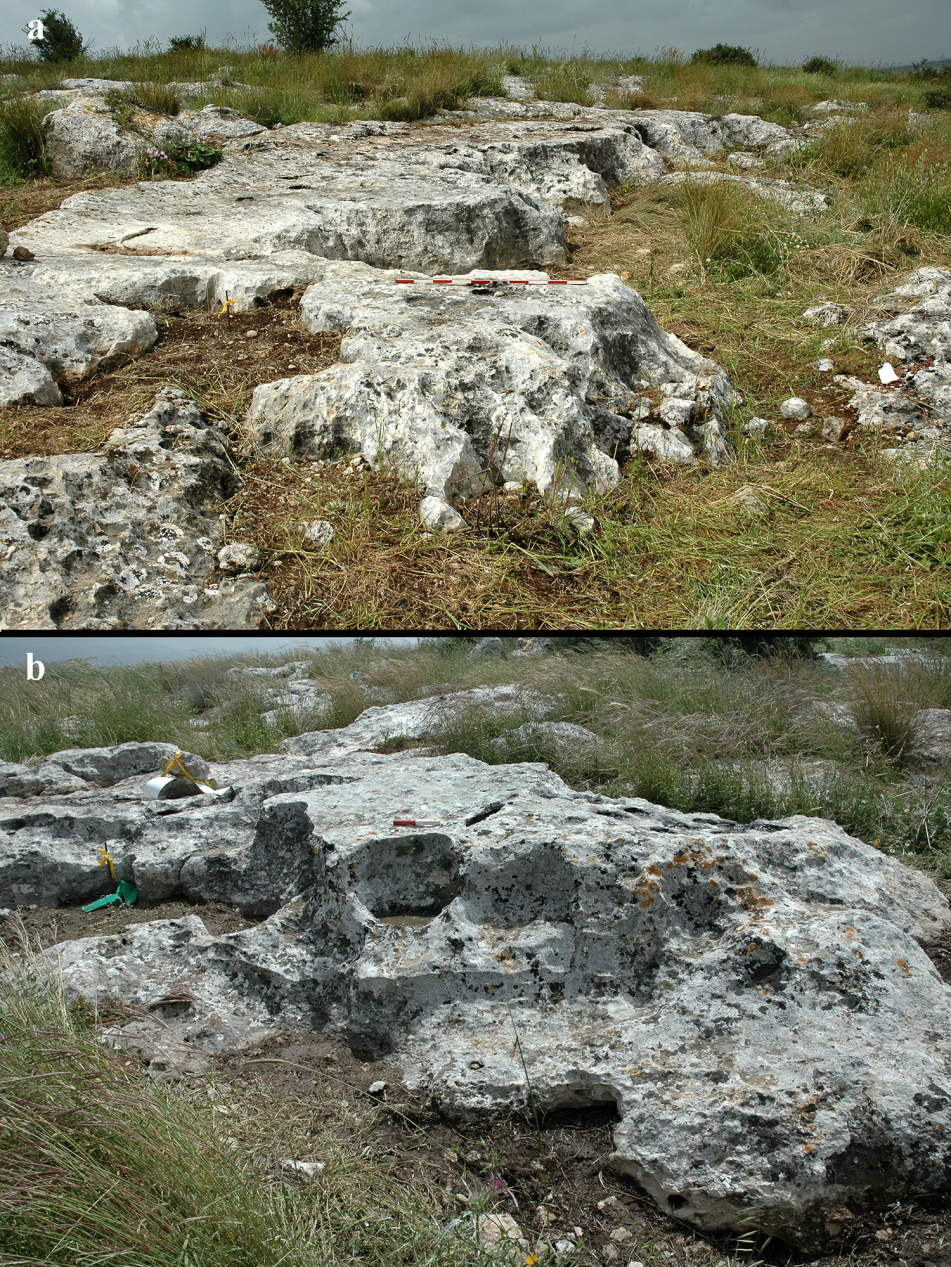
Quarrying fronts: a) general view of Rock #2; b) detailed view of Rock #3; note the step-like morphology of the quarrying front.

The flat *caliche* surfaces do not have any systematic geometric form and their edges are non-linear in plan view (Fig 14). We explain this lack of systematic geometry as resulting from the unpredictable location of the Meshash flint blocks and nodules in the *caliche*. The presence of extensive rock damage markings, and the multiple types of markings identified on the sloping and vertical edges of the surfaces, illustrate the extensive effort invested in quarrying. Once a large block of flint was extracted, it re-formed the edge of the quarrying front and hence dictated a new geometry of its edges. In cases where several large caches of flint were extracted in close proximity, each of the caches left a large void and the edge, after extraction, took on a very wavy morphology (Fig 15A and 15B). The rocks are cut by a series of 2–3 removals forming a step-like morphology in the *caliche* from the uppermost surface of the rock to its base (Fig 15B). These quarrying fronts are very different in character from those encountered on the sloping terraces of Kaizer Hill, where they are highly linear, forming straight lines and forming the terraces that are so characteristic of the topographically lower part of the hill [19, 20].

**Fig 16:**
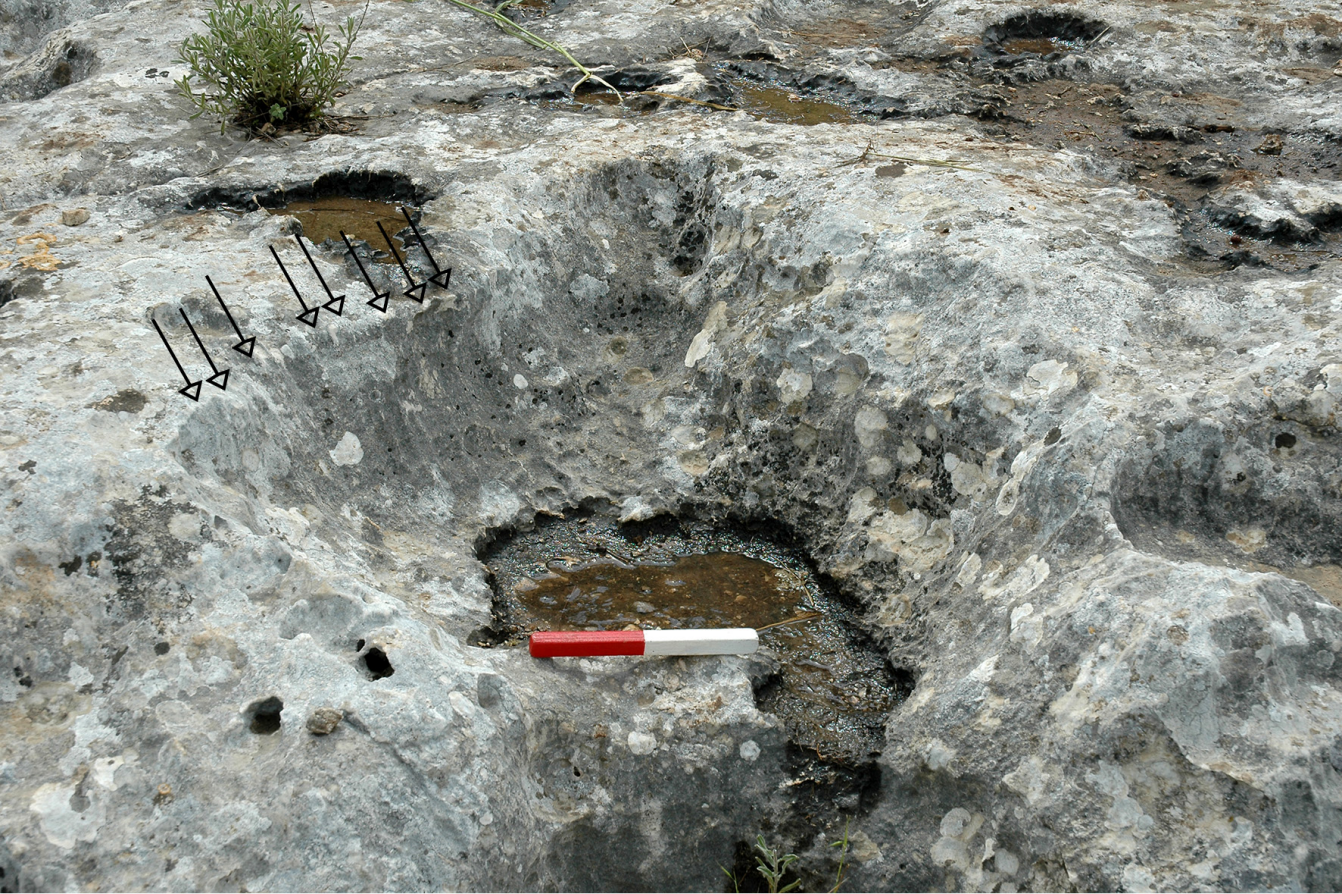
Parallel strips (note the arrows) of rock damage caused by a tool with a wide working edge.

Numerous rock damage markings were observed on the quarrying fronts. These include cupmarks of different types, drilling marks and marks that seem to have resulted from the use of a hard tool with a wide working edge (Figs 7B and 16).

The parallel strips of rock damage (2–3 cm wide) on the quarried walls are apparently traces left by the quarrying tools, most probably the Neolithic bifacial tools (axes and adzes) that were found in high frequencies at the site. One should, however, bear in mind that the Neolithic tool kit included a variety of other tools that may have left these markings. The composition of the different types of rock damage markings is not surprising, as they occur on the flat rock surfaces as well. The use of different tools indicates highly skilled craftsmanship capable of adjusting different tools to the complex task at hand.

The study of the quarrying fronts also resulted in our understanding that a particular method was involved in the process of quarrying. This is revealed by a series of steps, beginning at the top of the rock surface and descending (e.g, Fig 15B). This method was encountered on all three sampled rocks, but is also visible on other *caliche* surfaces of the Hilltop. We interpret this phenomenon as resulting from a particular strategy of progress during quarrying, which involved first extracting the nodules close to the surface, and then advancing toward the lower parts of the slope/quarrying front.

### The Lithic Assemblage

**Fig 17:**
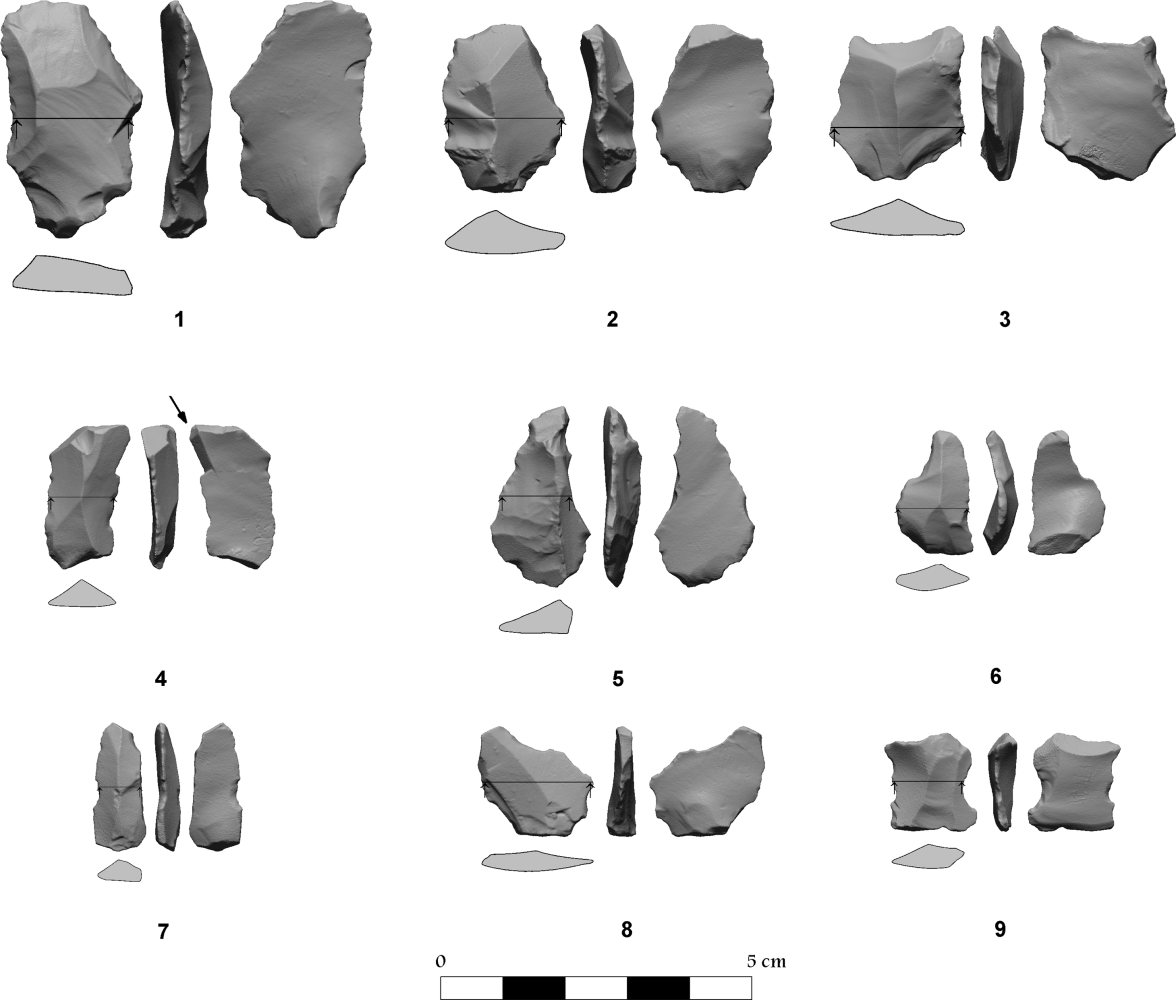
Flint tools: 1–3 retouched tools; 4 burin; 5–6 awls; 7–9 notched tools.

**Fig 18:**
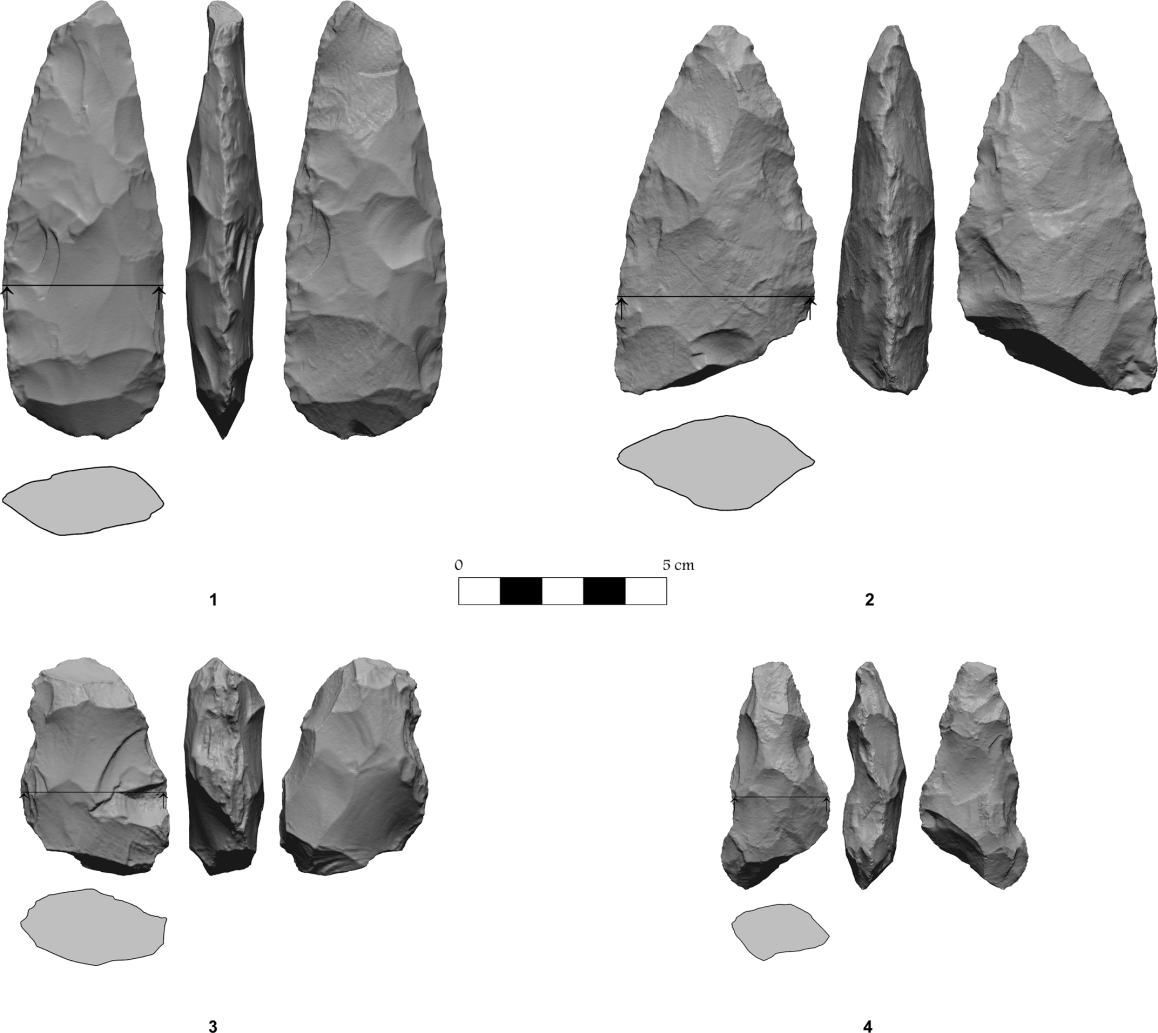
Flint bifaces.

The lithic assemblages collected in the periphery of the three rocks and the surveyed area were combined (N = 3,632) for the purposes of analysis (Table 2; Figs 17 and 18). As this was a surface collection, no sieving took place, and hence microartifacts and microlithic tools may be underrepresented in this assemblage (Table 2).

**Table 2:** The typology and frequencies of the lithic assemblage of Kaizer Hilltop.

Type	N	%
End-scrapers	11	3.5
Burins	19	6.1
Borers and awls	23	7.3
Backed pieces	15	4.8
Truncations	16	5.1
Notches and denticulates	65	20.7
Retouched pieces	96	30.6
Multiple tools	6	1.9
Non-geometric microlithics	9	2.9
Bifaces	47	15.0
Hammerstones	3	1.0
Varia	4	1.3
***Total Tools***	***314***	***100***
***Use Signs***	***163***	***100***
Flakes	650	65.2
Primary flakes	132	13.2
Blades/Bladelets	100	10.0
Primary blades/Bladelets	4	0.4
Ridge blade	4	0.4
Other CTE	102	10.2
Burin spall	2	0.2
Bifacial spall	3	0.3
***Total Débitage***	***997***	***100***
Chips	1703	81.3
Chunks	391	18.7
***Total Debris***	***2094***	***100***
Tool	314	8.6
Signs of use	163	4.5
Débitage	997	27.5
Cores	63	1.7
Debris	2094	57.7
***Total***	***3632***	***100***
***Tools/Cores***		***5*.*0***
***Débitage/Cores***		***15*.*8***
***Débitage/Tools***		***3*.*2***
***CTE/Core***		***1*.*7***
***Blades/Flakes (débitage)***		***0*.*1***
***Blades/Flakes (tools)***		***0*.*2***

The raw material most frequently used for the production of the different tool categories is a fine-grained gray flint (40.1%), while the typical brecciated Meshash Formation flint was used for making 24.5% of the tools, primarily large, massive tools like bifaces and hammerstones. Since the flint embedded in the *caliche* bedrock is usually devoid of calcareous cortex, artifacts bear remains of cortex in low frequencies (7.1%; N = 279). A high percentage of the artifacts, particularly débitage items, are broken (46%).

In total, more than half of the artifacts are debris and a third are débitage items (Table 2). The waste items are morphologically dominated by deep scar removals with pronounced bulbs of percussion. Among the débitage artifacts, 13.16% show signs of use wear, i.e., sheen and weathering of the scar ridges. Flakes are the most common débitage artifacts (78.4%) and also the most common blank for shaping tools (62% of tools), while blades/bladelets are much less common (débitage 10.4% and tools 16.6%). The cores represent 1.6% (N = 63) of the assemblage and most have a single striking platform (53%).

During the initial sorting of waste and tools, we identified a large group of artifacts that were slightly retouched (less than 1 cm of continuous retouch along the edge) or notched and hence could not be assigned to a particular tool category. Nevertheless, they represent a feature of the assemblage and were therefore separated and included under the category of signs of use (Table 2; Fig 17). This category represents half of the tools and 4.5% (N = 183) of the total assemblage. Both tools and signs of use comprise 6% of the assemblage. Worth noting are the “ad hoc” tools, which form the most abundant group of artifacts among the tool categories (retouched pieces: 30.6%; notches and denticulates: 20.7%). Clearly, if we combine the artifacts with signs of use and the “ad-hoc” tools, they are by far the most abundant tool category at the site. This fits well with the suggested nature of the assemblage, i.e., they are primarily a product of quarrying flint and the quarrying tools themselves.

The assemblage lacks projectile points and is characterized by very low frequencies of backed pieces, particularly sickle blades. Thus, the typical PPNA tools are missing from the Kaizer Hilltop lithic assemblage, and very few tools are specifically indicative of this chrono-cultural phase.

The most frequent “formal” tool is the biface (14.9%), although many of these (40.4%) show bifacial retouch but are not sufficiently standardized to be assigned to a sub-category of the bifaces (Fig 18). The other tools are divided into axes at a relative high frequency (48.5%), chisels (6.4%) and adzes (4.3%).

A 3D analysis of the bifaces from Kaizer Hilltop is ongoing; however, preliminary results can be summarized as follows:

- A high percentage (45%) of the bifaces are broken.
- The bifaces are roughly modified and substantially smaller than bifaces from other PPNA sites.
- The analysis of scar patterns of bifaces suggests that there was extensive postproduction damage (the analysis is using a technique previously used for postdepositional quantification; see [31]).
- The frequency of bifaces at Kaizer Hilltop is considerably higher than that reported from other PPNA large occupation sites such as Netiv Hagdud [32].

In sum, the characteristics of the lithic assemblage call for its assignment to the PPNA. Furthermore, we view this assemblage as a product of the quarrying activities. This is based on the high frequency of waste and “ad-hoc” tools, broken flakes, unstandardized flakes and very few “formal” tools. The paucity of arrowheads and sickle blades in this context is supportive of such a view. The abundance of bifaces and their characteristics suggest that they were used primarily as quarrying tools.

## Discussion and Summary

The varied types of rock damage observed on the *caliche* rock surfaces of Kaizer Hilltop were recorded and then extensively analyzed. We present below interpretations and conclusions that go beyond the site.

The quarrying fronts of the Hilltop are distinctive, and they have many and varied shapes (contours) that include straight, concave and convex morpholines. It is evident that the particular layouts of the quarrying fronts are dictated by the presence, form, size and quantities of flint blocks/nodules of the Meshash Formation, but above all by their location within the *caliche* crust. In places one can observe rock depressions that were formed by the extraction of flint (e.g., Figs 10, 12, 15 and 16).

Current investigations of the Hilltop have identified only a few waste piles. This lack could have been due to the nature of the *caliche*: after it was quarried and broken into small fragments, it may have been subjected to postdepositional processes, mainly weathering agents and transportation down the slope towards the wadi. In contrast, and in close proximity, many waste piles were identified and studied at the quarrying fronts of the terraced slopes built of hard limestone of the Bina Formation [20].

In addition to the quarrying fronts, there are extensive rock damage signs that demonstrate that the fronts were only one expression of the quarrying activities. The flat top surfaces of the *caliche* are spotted with different markings, most frequently by cupmarks of various sizes and shapes. Less common are trenching or isolated drilling marks.

We interpret the trenching/drilling observed on the Kaizer Hilltop as a particular strategy applied to search for flint. This strategy seems to have been a combined one, incorporating a search of the top surface of the *caliche*, drilling into its depth, and then, once the cache of flint was detected, the opening of an extensive quarry front in which quarrying advanced through the full thickness of the *caliche*. The drilling of trenches was a component of the general strategy of exhausting the potential of unknown flint caches in the *caliche*. All sampled rock surfaces and many others were divided into several sub-surfaces by drillings that started at the rock surfaces. This activity divided the rock surfaces into several segments and hence allowed easy access to the *caliche* from additional vertical exposures, thus increasing the visibility of additional flint caches.

The end result of such a quarrying process is expressed by isolated *caliche* surfaces whose edges are exposed vertically down to the bottom of the *caliche* (the underlying soft bedrock of the Menuha Formation).

The only material culture remains are lithic artifacts, while ground stone tools, faunal remains, etc., are absent. In its characteristics the lithic assemblage conforms with the nature of a task-specific site–a quarry. Apparently, the tools most often utilized during quarrying were drills (borers and awls) and flint artifacts of other types. The high frequency of quarrying marks reflecting the use of bifaces, and the abundance of actual bifaces (axes and adzes) in the lithic assemblage, suggest that these artifacts were quarrying tools.

Although this is an unconventional suggestion in the Levantine Neolithic context, as most researchers view bifacial tools as artifacts designed for woodworking (e.g., [33]), our interpretation is worth testing, particularly since the *herminette* (a unifacial flint tool) was used at Jerf el Ahmar, Syria, to modify soft limestone blocks [34]. For several other archaeological sites it has been suggested that the biface was the quarrying tool. At ‘Ain Ghazal there are clear indications of quarrying although the quarrying tool is yet to be identified; still, high frequencies of bifaces suggest that it might have been the quarrying tool [35]. Another example is the basalt axes retrieved from the Italian site of Monte Tabuto [36], which were considered to be the quarrying tool. Lastly, at the quarry site of Jabilens high percentages of bifacial tools were found [37]. There are many other European Neolithic sites in which bifacial tools are the main quarrying artifacts. Future studies of the Kaizer Hill assemblage will discuss the role of the bifacial tools in detail.

Quarrying techniques at both Kazier Hilltop and Hatula [5] left their damage markings in the form of round cupmarks as a result of opening the *caliche* surface. Penetrating through the extremely thin top layer of the *caliche* that seals the soft and powdery rock enabled access to the flint caches. At Kaizer Hilltop quarrying fronts were formed by a combination of a series of cupmarks chiseled into the *caliche*, cutting into each other and leaving at the base the negative of the targeted raw flint nodule. Similar fronts are less frequent at Hatula, where it seems that modern alluvial soil covers most of the vertical faces of the rock surfaces. What is most surprising is that different tools were utilized at both sites. While at Hatula the main activity was drilling, at Kaizer Hilltop it was chiseling. Finally, the morphology of the quarrying fronts at Kaizer Hilltop rules out the possibility that the *caliche* was exploited for the production of slabs, as was the case at Hatula [5].

We suspect that Hatula and Kaizer Hilltop are the tip of the iceberg with respect to quarry sites on the series of hills situated between the Shephelah and the Judean Hills. Many sites in this small geographical region have been culturally assigned to the PPNA (Fig 2). Most sites are located on limestone bedrock surfaces, accompanied by Cenomanian and Senonian flint outcrops without architectural features [16]. An illuminative example is the PPNA site of Tzur Natan, where many rock cupmarks were reported (Fig 2, [16]); there are also many similarities between the lithic assemblages of this site and those of Kaizer Hilltop. The Tzur Natal lithics are poor in diagnostic PPNA tools (e.g., arrowheads, sickle blades) and the industry is primarily focused on flake production. The most common tools are mainly “ad hoc” types (retouched flakes, notches and denticulates, retouched blades and scrapers), while the dominant formal tool is the biface.

In conclusion, a variety of settlement types is present in the region, the differences among them probably reflecting task-specific activities. We assume that once drilling and chiseling had become a common technology of the Neolithic society of the southern Levant, it was used for many other tasks.

At Kaizer Hilltop large-scale quarrying was carried out in the *caliche* to obtain flint. We have demonstrated the unique properties of this quarry site and its varied rock damage. There is no doubt that the PPNA occupants of the area changed the landscape forever, extensively “peeling” the *caliche* surfaces in search of the flint imbedded in the calcareous bedrock.

An ongoing research program aims to test other questions that were formulated during the survey at the Kaizer Hill quarry site. These are concerned with the quarrying strategies and techniques employed by the PPNA people on the terraced limestone slopes, i.e., their organized waste management system and how it differs from the *caliche* extractions. Future studies will enable a comparison between the quarrying damage types observed on the Hilltop, where soft bedrock prevails, and those encountered in the hard limestone Bina Formation. Only then will we be able to present a detailed picture of the strategy and tactics that were employed by the PPNA quarrying groups at Kaizer Hill.
